# Editorial: The wellbeing of criminal justice personnel

**DOI:** 10.3389/fpsyg.2023.1245541

**Published:** 2023-07-13

**Authors:** Andrew James Clements, Jessica Woodhams, Joseph K. Young, Fazeelat Duran

**Affiliations:** ^1^Aston Business School, Aston University, Birmingham, United Kingdom; ^2^School of Psychology, University of Birmingham, Birmingham, United Kingdom; ^3^School of Public Affairs and the School of International Service, American University, Washington, DC, United States

**Keywords:** wellbeing, criminal justice personnel, trauma, child sexual abuse and exploitation, shift-work, mental health stigma

Criminal Justice Personnel (CJP) in policing, security, and justice organizations are exposed to a variety of hazards. These hazards include negative interactions with members of the public, e.g., due to distrust (Adams and Buck, 2010; Can et al., 2018), and interactions with distressed individuals (Schrever et al., 2022). They may encounter aggressive behaviors including intimidation (Kinman and Clements, 2022) and violence (Ellrich, 2016; Isenhardt and Hostettler, 2020). There is also potential for exposure to traumatic content, e.g., through investigation of offenses involving violence or abuse (Duran and Woodhams, 2022). They may encounter scenes of violence and death at crime scenes (Salinas and Webb, 2018) and encounter prisoner self-harm and suicide (Marzano et al., 2015). There are organizational hazards too (Clements et al., 2021), such as unsocial working hours (Hu et al., 2015; Scholarios et al., 2017), and under-resourcing (Duran, 2019), e.g., short-staffing (Martin et al., 2012; Kinman et al., 2019). CJP are therefore at risk of experiencing psychological ill-health (Warren et al., 2015; Magnavita et al., 2018; Foley and Massey, 2019; Clements and Kinman, 2021; Allison et al., 2022). This Research Topic explores the wellbeing challenges of CJP, with a particular focus on potentially traumatic content.

Three papers report research on the wellbeing of those involved in investigations of child sexual abuse and exploitation (CSAE) (Redmond et al.; Simonovska et al.; Strickland et al.). Simonovska et al. recruited an international sample of current (*N* = 516) and former (*N* = 126) personnel of CSAE units. They reported that participants were most concerned by work pressure and inadequate resources. However, former personnel also frequently identified exposure to CSAE material as having impacts, leading the authors to suggest the impact of exposure may make itself known over a longer period. Simonovska et al. further found that both current and former personnel reported developing a more negative worldview and becoming more protective of children due to their work. Positively, participants identified resources that helped them in their work, such as humor-based coping, seeing the results of their efforts, and support from colleagues.

Strickland et al. conducted interviews with seven digital forensic specialists and analyzed the data using interpretive phenomenological analysis. Their findings suggested that participants experienced pervasive changes to their worldview, perceiving abuse more readily. Strickland et al. further reported participants' active efforts to maintain work-life boundaries, e.g., through exercise or using commute time to decompress. While their participants often reported positive relations with line managers, there were indications of organization cultural barriers to seeking support, e.g., mental health stigma.

Redmond et al. report a survey of police officers and staff regularly dealing with CSAE (*N* = 661) on their help-seeking behaviors. Some of the barriers they identified included a lack of trust in the organization (e.g., that mental health challenges would remain confidential), a perception that some processes (including some support available) were a “tick box exercise,” and a perception that individuals were expected to cope alone. Redmond et al., similar to some scholars, argue that the organizational context may have more impact than the work itself (i.e., CSAE).

Studies in this Research Topic have also considered other criminal justice professions. One brief research report examines suicidal ideation in corrections workers (Johnston and Ricciardelli). Johnston and Ricciardelli gathered qualitative data from 94 employees in Canada, using data from open-ended survey items. In keeping with other research in this Research Topic, participants highlighted the role of high work demands, perceptions of a lack of support, and a reluctance to seek help due to stigma surrounding suicide. The authors argue that employers are in a difficult position, given that offers of support may be seen as failing to address underlying issues (e.g., workload) and that individuals may decline to seek support due to suspicions of the organization providing support and concerns relating to stigma.

A further study focuses on the experiences of Crown prosecutors, who deal with more serious cases in New Zealand (Kim et al.). Kim et al. conducted semi-structured interviews with 19 practicing Crown prosecutors about their experience of working with potentially traumatic material (PTM). Their participants reported encountering PTM in a variety of forms relating to evidence, which could include being present at post-mortems. Like other studies in this issue, the Crown prosecutors experienced pervasive changes to their perception of life because of their work. In managing PTM, there were signs that some participants saw becoming “detached” and “clinical” as desirable, although this was not a unanimous view.

Given the themes emerging from the studies above, it is promising to see that there are signs of attention to interventions. One paper in this issue reports interview data collected during a feasibility study on the use of psychological first aid (PFA) in policing (Geoffrion et al.). Geoffrion et al. interviewed 26 PFA responders, 4 recipients of PFA, and 6 managers. Broadly, participants spoke positively about the programme making it support more accessible, being proactive, and that it was provided by peers. However, participants noted that because PFA providers were supervisors, willingness to access support might be influenced by the quality of working relationships, and desires to maintain positive impressions.

CJP are exposed to organizational stressors such as unsociable hours. The final paper in this issue examines the impact of policing shift-work on aspects of wellbeing (James et al.). James et al. gathered cross sectional data from 319 municipal police employees. Compared with day- and evening-shift participants, those working night shifts reported significantly worse sleep quality, higher levels of sleepiness, and greater likelihood of falling asleep while driving. However, James et al. found no effect of shift pattern on PTSD, depression, anxiety, nor quality of life.

Taken together, this Research Topic contributes to the developing evidence base on CJP wellbeing, with a particular focus on direct and indirect exposure to traumatic experiences of other people. There is a clear need for targeted support which accounts for barriers to access (e.g., stigma). We urge organizations, practitioners, and researchers to direct efforts toward development and evaluation of interventions.

## Author contributions

AC lead in writing this editorial. JW, JY, and FD contributed to revising drafts of the editorial. All authors contributed to the article and approved the submitted version.
